# Efficacy of mind–body exercise for perinatal depression and anxiety: a systematic review and meta-analysis

**DOI:** 10.3389/fpubh.2025.1709845

**Published:** 2025-11-11

**Authors:** Meng Liu, Mingyu Liao, Jiaran Jiang, Xueqiang Zhu, Keyin Liu

**Affiliations:** 1 School of Sport, Nanning Normal University, Nanning, China; 2 Department of Physical Training, Institute of Aviation Safety and Security, China Civil Aviation Flight Academy, Chengdu, China; 3 Department of General Education, Shandong Xiehe University, Jinan, China; 4 School of Competitive Sport, Shandong Sport University, Jinan, China

**Keywords:** mind–body exercise, perinatal, depression, anxiety, meta-analysis

## Abstract

**Background and objective:**

Although mind–body exercise is a promising non-pharmacological intervention, its overall efficacy for perinatal depression and anxiety remains unclear due to a lack of comprehensive assessment.

**Methods:**

Multiple databases were systematically searched to identify randomized controlled trials (RCTs) of mind–body exercise interventions for depressive and anxiety symptoms in perinatal women. A total of 13 studies were ultimately included. A meta-analysis was conducted to synthesize the effect sizes, and the GRADE methodology was used to assess the quality of evidence.

**Results:**

The meta-analysis revealed that mind–body exercise significantly improved both depression (SMD = −1.30, 95% CI: −1.86 to −0.73) and anxiety symptoms (SMD = −1.15, 95% CI: −1.84 to −0.45). However, there was extremely high heterogeneity among the studies (*I*^2^ > 93%), and the GRADE evidence quality was “very low.” Subgroup analyses indicated that the improvement in depressive symptoms was associated with the duration, period, and frequency of the intervention.

**Conclusion:**

Mind–body exercise may be beneficial for improving perinatal depression and anxiety, but the current evidence is of very low quality and high heterogeneity. Future research should focus on conducting large-sample RCTs with more rigorous designs and standardized reporting to provide more reliable evidence.

## 1 Introduction

The perinatal period, a critical stage in a woman’s life, is generally defined as the time from pregnancy to 1 year postpartum (1). During this period, women face complex psychological changes that can have a profound impact on their mental health. Perinatal depression (PND), which includes depressive symptoms occurring during pregnancy (antenatal depression) or after childbirth (postpartum depression), is a common psychiatric disorder (2, 3). The World Health Organization (WHO) reports that globally, approximately 10% of pregnant women and 13% of new mothers experience a mental disorder, primarily depression. However, this burden is unevenly distributed worldwide, with proportions rising significantly in low- and middle-income countries (LMICs), where the prevalence is as high as 15.6% during pregnancy and 19.8% postpartum. A recent systematic review and meta-analysis, synthesizing 4,242 primary studies, revealed an even more severe situation, finding a global pooled mean prevalence of perinatal depression of 26.3% (4). The occurrence of perinatal anxiety symptoms also warrants attention (5, 6). A systematic review showed that up to 24.6% of women report anxiety symptoms in late pregnancy, and the prevalence of clinically diagnosed anxiety disorders throughout the perinatal period can reach 15.2% (7). Mental health problems not only affect the mother’s quality of life and family harmony but can also have adverse long-term effects on fetal and infant development (8).

In response to this issue, mind–body interventions (MBIs) have gained increasing attention as a non-pharmacological approach. These interventions, such as yoga, mindfulness, and Tai Chi, focus on regulating the interplay between the brain, mind, body, and behavior through specific activities to enhance an individual’s self-regulatory capacity over mind–body functions (9). In recent years, several studies have provided initial evidence of their potential (10). Research has shown that prenatal yoga can significantly improve depressive and anxiety symptoms during pregnancy and may reduce the risk of postpartum depression (11); mindfulness interventions have demonstrated positive effects in reducing perinatal stress and improving overall mental well-being (12, 13). Furthermore, with technological advancements, digital mind–body interventions (eMBIs) delivered via mobile health (mHealth) platforms have rapidly emerged. They overcome geographical and time constraints, offering new possibilities for providing low-cost, scalable mental health support, especially in resource-limited settings (14, 15). However, current research findings are inconsistent. Some studies have found no significant difference between MBI groups and control groups, with limited effects in certain populations. For instance, some systematic reviews on mindfulness interventions have reported small effect sizes and insufficient evidence for long-term efficacy (16).

At the same time, existing research has significant limitations. First, published systematic reviews often focus on a single form of mind–body exercise, such as meta-analyses specifically on yoga or mindfulness meditation, lacking a comprehensive evaluation of mind–body exercise as a broad intervention category. Second, most meta-analyses concentrate only on depressive symptoms, with relatively less attention paid to anxiety symptoms (17). Additionally, the methodological quality of existing studies is variable, with some having small sample sizes, unclear randomization methods, and difficulties in implementing blinding, which compromises the reliability of the evidence (18). Finally, although individual studies have shown positive results, there is a lack of high-quality traditional meta-analyses to quantify the comprehensive effect size of mind–body exercise on perinatal depression and anxiety, limiting the evidence base for clinical decision-making.

Therefore, this study aims to be the first to conduct a comprehensive quantitative assessment of the overall effectiveness of mind–body exercise as a broad intervention category in improving depressive and anxiety symptoms in perinatal women. First, by integrating the evidence from existing RCTs, we will quantify the overall pooled effect size of mind–body exercise on perinatal depression and anxiety symptoms. Second, through pre-specified subgroup analyses and meta-regression, we will systematically explore potential effect moderators to explain the high heterogeneity among studies. Finally, we will use the Grading of Recommendations Assessment, Development and Evaluation (GRADE) approach to assess the overall quality of the evidence. The results of this study will provide clinicians, policymakers, and perinatal women with a clearer and more reliable evidence-based summary of the effectiveness of mind–body exercise, thereby guiding clinical practice, optimizing intervention protocols, and indicating directions for future high-quality research.

## 2 Methods

This study was conducted following the Preferred Reporting Items for Systematic Reviews and Meta-Analyses (PRISMA) (19) and the Cochrane Handbook guidelines. The protocol has been registered with the International Prospective Register of Systematic Reviews (PROSPERO).

### 2.1 Literature search and study selection

To identify relevant studies evaluating the effectiveness of mind–body exercise on perinatal mental health outcomes, we conducted a systematic search of the following electronic databases: Cochrane Library, PubMed, Embase, Web of Science, and CINAHL. The search covered the period from the inception of each database to September 1, 2025 (Table 1).

**Table 1:** PubMed database literature search strategy.

Step	Search terms	Field
1	Pregnan OR prenatal OR antenatal OR maternal OR postpartum OR postnatal OR perinatal OR (expectant mother)	Title, abstract
2	(Mind body) OR mindfulness OR meditation OR yoga OR (tai chi) OR qigong OR pilates OR (mindful movement) OR (contemplative practice)	Title, abstract
3	Depression OR (postpartum depression) OR (perinatal depression) OR anxiety OR (perinatal anxiety) OR GAD OR (GAD-7) OR EPDS	Title, abstract
4	(Randomized controlled trial) OR randomized OR randomly OR trial OR (control group) OR RCT	Title, abstract
5	#1 AND #2 AND #3 AND #4	

### 2.2 Study selection criteria

Inclusion and exclusion criteria were established based on the PICOS (Population, Intervention, Comparison, Outcome, Study Design) framework. The specific selection criteria are detailed in Table 2.

**Table 2:** Inclusion and exclusion criteria for literature.

PICOS components	Inclusion criteria	Exclusion criteria
Population	Perinatal women (including pregnancy and up to 1 year postpartum), age ≥18 years, and singleton or multiple pregnancies.	Adolescents aged <18 years, individuals with a history of severe physical or psychiatric disorders, those with pregnancy complications (e.g., preeclampsia, gestational diabetes), and those with a history of substance or alcohol abuse.
Intervention	Mind–body therapies (yoga, meditation, mindfulness, Tai Chi, qigong, Pilates, etc.), intervention duration ≥4 weeks, and clear description of intervention protocol.	Pharmacological treatment only, traditional psychotherapy (e.g., CBT), intervention duration <4 weeks, and unclear intervention protocol description.
Comparison	Usual prenatal/postnatal care, waitlist control, placebo control, or other activity control.	Studies without control groups, historical controls, and control groups receiving concurrent psychological
Outcomes	Depression symptom scores (e.g., EPDS, PHQ-9, BDI), anxiety symptom scores (e.g., GAD-7, STAI, BAI).	Reporting only physiological indicators, no standardized mental health assessment tools, and unclear outcome measures or extractable data unavailable.
Study design	Randomized controlled trials (RCTs) published in peer-reviewed journals and providing complete study data.	Observational studies, case reports, reviews or meta-analyses, conference abstracts or grey literature, and incomplete data or full-text unavailable.

### 2.3 Data extraction and preparation

A customized data extraction form was developed in Microsoft Excel (Microsoft Inc., Redmond, WA, United States). Reference management and deduplication were performed using EndNote software. Following an initial automatic deduplication, one researcher manually screened the records to identify and remove any remaining duplicates. Two reviewers independently extracted data from the included studies; the extracted data were then cross-checked for accuracy and completeness. Any discrepancies were resolved through discussion or, if necessary, by consulting a third reviewer. The extracted variables included: first author, publication year, sample size, participant age, intervention details, gestational stage, intervention duration, outcome measures, and assessment tools. All data were double-entered and verified to minimize data entry errors and enhance the reliability of the review.

### 2.4 Study quality assessment

Two authors (MeL and MiL) independently assessed the methodological quality of all included studies. For randomized controlled trials (RCTs), the Cochrane risk-of-bias tool was employed, evaluating the following seven domains: (1) random sequence generation (selection bias); (2) allocation concealment (selection bias); (3) blinding of participants and personnel (performance bias); (4) blinding of outcome assessment (detection bias); (5) incomplete outcome data (attrition bias); (6) selective reporting (reporting bias); and (7) other sources of bias. The risk of bias for each domain was categorized as “low risk,” “unclear risk,” or “high risk.” The overall risk of bias for each study was classified as “low risk” (low risk across all key domains), “moderate risk” (unclear risk in one or more key domains but no high-risk domains), or “high risk” (high risk in one or more key domains). Disagreements between reviewers were resolved by consensus; if consensus could not be reached, a third author (XZ) made the final determination.

### 2.5 Statistical analysis

A meta-analysis was planned to estimate the pooled effect of mind–body exercise on perinatal depression and anxiety. All statistical analyses were conducted using Review Manager (Version 5.4). Effect sizes were calculated as mean differences (MD) when studies used the same outcome measure, or as standardized mean differences (SMD) when different measures were used, to allow for comparability. A random-effects model was pre-specified for pooling data if substantial heterogeneity was detected (*I*^2^ > 50%), while a fixed-effect model would be used for low heterogeneity (*I*^2^ < 50%). Where heterogeneity was high, subgroup analyses were planned to investigate potential sources of variation. A leave-one-out sensitivity analysis was planned to assess the influence of individual studies on the overall pooled estimate. Furthermore, univariate meta-regression analyses were planned for pre-specified moderators to explore potential effect modification. Based on theoretical considerations and prior evidence, we pre-specified subgroup analyses for three key moderating variables: (1) Intervention duration: a systematic review of internet-based interventions found that longer programs (>8 weeks) were more effective for depression and anxiety (20). As the median duration for psychological interventions in a previous meta-analysis was 9 weeks (21), we used an 8-week threshold to explore its potential moderating effect. (2) Intervention frequency: a meta-analysis by Tiemens et al. demonstrated that a higher session frequency during the initial months of treatment for depression was associated with better outcomes (22). (3) Session duration: research on brief psychotherapy indicates that 30-min sessions can be effective (23), while principles from cognitive psychology suggest that sustained attention wanes over time, potentially leading to cognitive fatigue in longer sessions (24). Therefore, this study used 30 min as a cutoff to explore the potential effect-modifying role of single-session duration.

## 3 Results

### 3.1 Literature search

**Figure 1:**
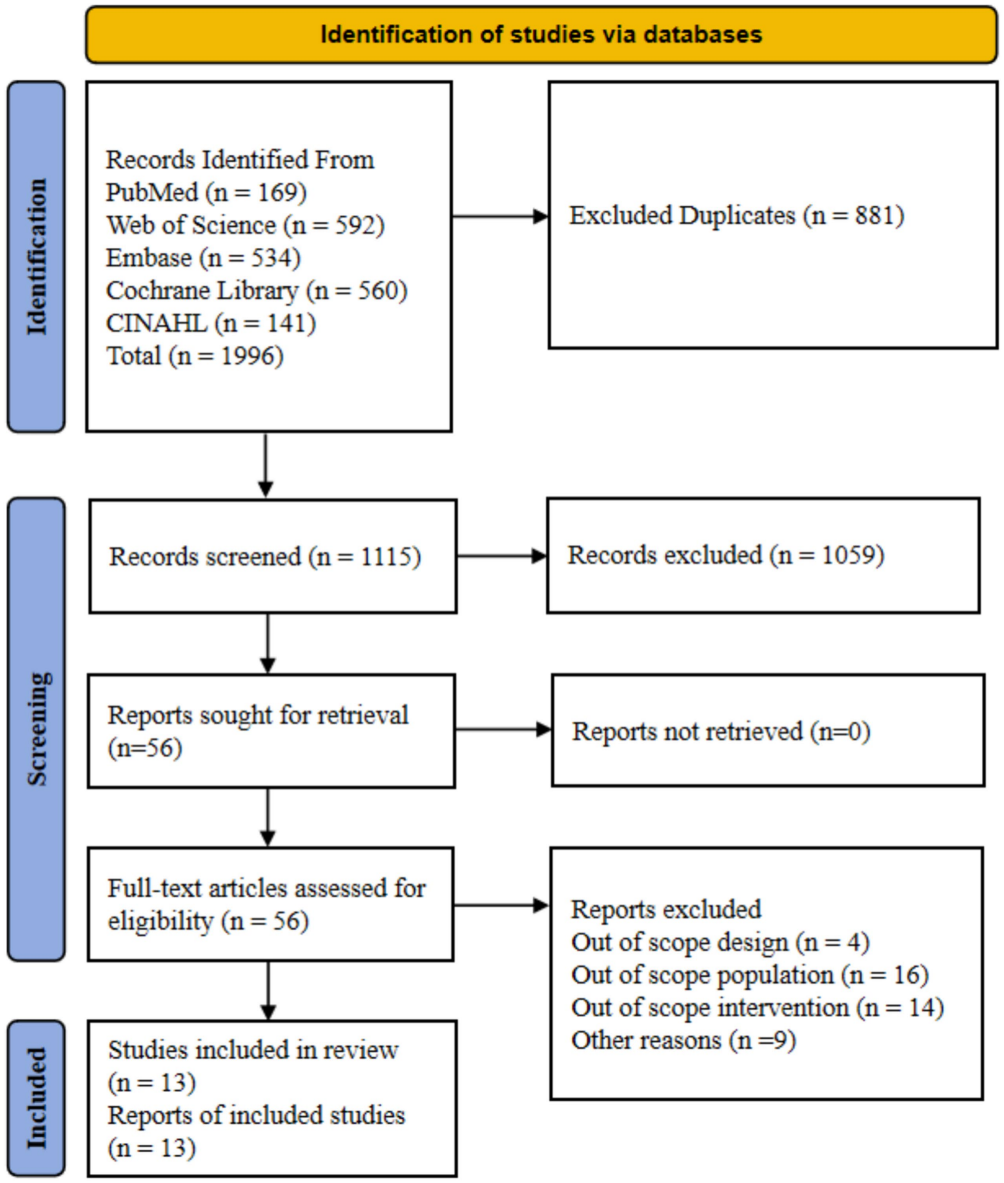
Preferred reporting items for systematic reviews and meta-analysis (PRISMA) study flow diagram.

The systematic search across PubMed, Web of Science, Embase, the Cochrane Library, and CINAHL initially yielded 1,996 records. After the removal of 881 duplicates, the titles and abstracts of the remaining 1,115 records were screened. From this, 1,059 records were excluded as they were thematically irrelevant, were not randomized controlled trials, or were not published in English. The full texts of the remaining 56 articles were retrieved and assessed for eligibility. Of these, 43 articles were subsequently excluded for reasons including non-conforming study design (*n* = 4), population (*n* = 16), intervention (*n* = 14), or other reasons (*n* = 9). Ultimately, 13 studies fulfilled all inclusion criteria and were included in the final systematic review and meta-analysis. The complete study selection process is illustrated in the PRISMA flow diagram (Figure 1).

### 3.2 Characteristics of included studies

This study included 13 randomized controlled trials (25–37), published between 2012 and 2025 (25, 37), with a total sample size of 1,052 perinatal women (Table 3). The sample size of individual studies ranged from 16 to 316 participants, with the smallest being 16 (29) and the largest being 316 (27). Regarding population characteristics, the average age of participants ranged from 24.4 to 39.7 years (29, 37), with most subjects being in the 20–30 age group. The distribution of study phases showed that 12 studies intervened with pregnant women, while only one study specifically targeted postpartum women (32). Analysis of intervention types revealed that yoga was the most widely used form of mind–body exercise, accounting for 8 studies, followed by mindfulness interventions in 3 studies (26, 27, 31), Pilates in 1 study (29), and one study using a Tai Chi/yoga intervention (35). There were significant variations in intervention parameters. The duration of interventions ranged from 4 to 16 weeks (26, 28, 36), with 8-week interventions being the most common (27, 29, 31–34), followed by 12-week interventions (30, 35, 37). The frequency of interventions varied from once a week to five times a week (28, 34), with 2–3 times per week being the most prevalent. The duration of a single session ranged from 20 to 180 min (31, 37), with most studies adopting a moderate duration of 40–90 min. In terms of outcome assessment, eight studies evaluated both depressive and anxiety symptoms (27, 28, 30, 32–35, 37), 4 studies assessed only depressive symptoms (25, 29, 31, 36), and 1 study assessed only anxiety symptoms (26). A variety of measurement tools were used; depression was primarily assessed using the EPDS and CES-D scales (29, 35), while anxiety was mainly assessed using the STAI and PRAQ-R scales.

**Table 3:** Basic characteristics of included studies.

Author, year	Mean age (years) (intervention/control)	Intervention (intervention/control)	Sample size (*n*) (intervention/control)	Perinatal stage	Duration (weeks)	Frequency	Session length (min)	Outcomes	Assessment tools
Kim and Hyun, 2022 (29)	39.71 ± 2.01/38.14 ± 1.39	Pilates vs. usual prenatal care	8/8	Mean gestational age (GA): 24–28 weeks	8	2x/week	50	Depression	EPDS
Gökbulut et al., 2024 (26)	28.5 ± 8/28 ± 3	Mindfulness vs. usual prenatal care	32/32	Mean GA: 12–24 weeks	4	2x/week	40–60	Anxiety	PRAQ-R2
Newham et al., 2014 (34)	31 ± 5/31 ± 7	Yoga vs. usual prenatal care	29/22	Mean GA: 20–24 weeks	8	1x/week	90	Depression, anxiety	EPDS, WDEQ
Pan et al., 2019 (31)	32.8 ± 3.9/33.8 ± 3.9	Mindfulness vs. usual prenatal care	39/35	Mean GA: 20.7 ± 4.8 weeks	8	1x/week	180	Depression	EPDS
Hassdenteufel et al., 2023 (27)	32.6 ± 4.3	Mindfulness vs. usual prenatal care	142/174	Mean GA: 16–20 weeks	8	1x/week	45	Depression, anxiety	EPDS, PRAQ-R
Nadholta et al., 2023 (28)	29.31 ± 3.41/29.71 ± 3.00	Yoga vs. usual prenatal care	34/43	Mean GA: 21.2 ± 4.3 weeks	16	5x/week	40–60	Depression, anxiety	DASS-42
Rong et al., 2021 (30)	29.00 ± 2.81/28.16 ± 2.78	Yoga vs. usual prenatal care	32/32	Mean GA: 22.44 ± 3.39 weeks	12	3x/week	60	Depression, anxiety	EPDS, S-AI
Field et al., 2013 (35)	24.4 ± 4.7/26.0 ± 5.6	Tai Chi Yoga vs. usual prenatal care	46/46	Mean GA: ~22 weeks	12	1x/week	20	Depression, anxiety	CES-D, STAI
Field et al., 2012 (37)	29 ± 2.81/28.16 ± 2.78	Yoga vs. usual prenatal care	32/32	Mean GA: 18–22 weeks	12	2x/week	20	Depression, anxiety	CES-D, STAI
Lee et al., 2025 (25)	32.63 ± 3.11/31.87 ± 4.46	Yoga vs. usual prenatal care	30/31	Mean GA: 20–26 weeks	12	3x/week	60	Depression	EPDS
Buttner et al., 2015 (32)	29.81 ± 5.17/32.45 ± 4.78	Yoga vs. usual prenatal care	27/29	Postpartum (mean: 4.63 ± 3.47 months)	8	2x/week	60	Depression	HDRS
Davis et al., 2015 (33)	29.74 ± 5.40/30.57 ± 4.46	Yoga vs. usual prenatal care	23/23	Mean GA: 20.78 ± 6.42 weeks	8	1x/week	75	Depression, anxiety	EPDS, STAI
Satyapriya et al., 2013 (36)	26.41 ± 3.01/24.96 ± 2.58	Yoga vs. usual prenatal care	51/45	Mean GA: 18–20 weeks	16	3x/week	60	Depression, anxiety	HDRS, STAI

### 3.3 Quality assessment of literature

**Figure 2:**
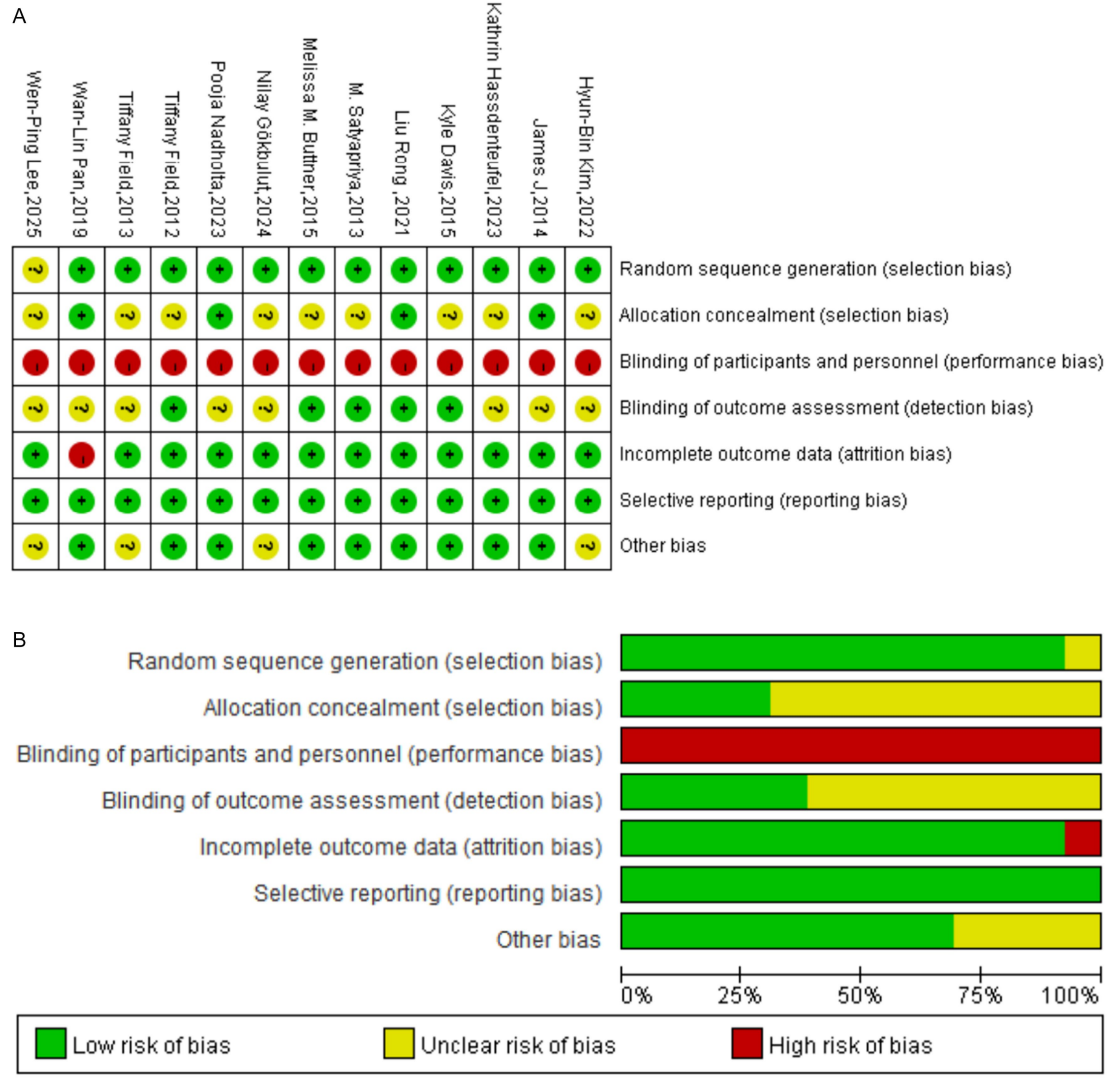
**(A)** Summary map of RCT bias analysis. **(B)** Graphical map of RCT bias analysis.

The methodological quality of the 13 included RCTs was assessed using the Cochrane risk-of-bias tool, with results summarized in Figures 2A,B. The assessment revealed the following: for random sequence generation, all 13 studies were rated at low risk of bias. For allocation concealment, four studies were at low risk, while nine were at unclear risk. A high risk of performance bias was evident across all studies due to the inherent difficulty of blinding participants and personnel to a mind–body intervention. For detection bias (blinding of outcome assessment), five studies were at low risk, and eight were at unclear risk. Regarding attrition bias (incomplete outcome data), 12 studies were at low risk, and one was at high risk. All studies were judged to be at low risk of reporting bias. For other potential sources of bias, nine studies were at low risk, and four were at unclear risk. Overall, the methodological quality of the included studies was deemed moderate. However, significant limitations were identified, stemming primarily from an unclear risk of bias for allocation concealment in the majority of studies (*n* = 9), a high risk of performance bias due to the lack of participant and personnel blinding (*n* = 13), and an unclear risk of detection bias in outcome assessment (*n* = 8). While blinding is challenging in trials of behavioral interventions, these limitations may nonetheless compromise the reliability of the findings.

### 3.4 Meta-analysis results

**Figure 3:**
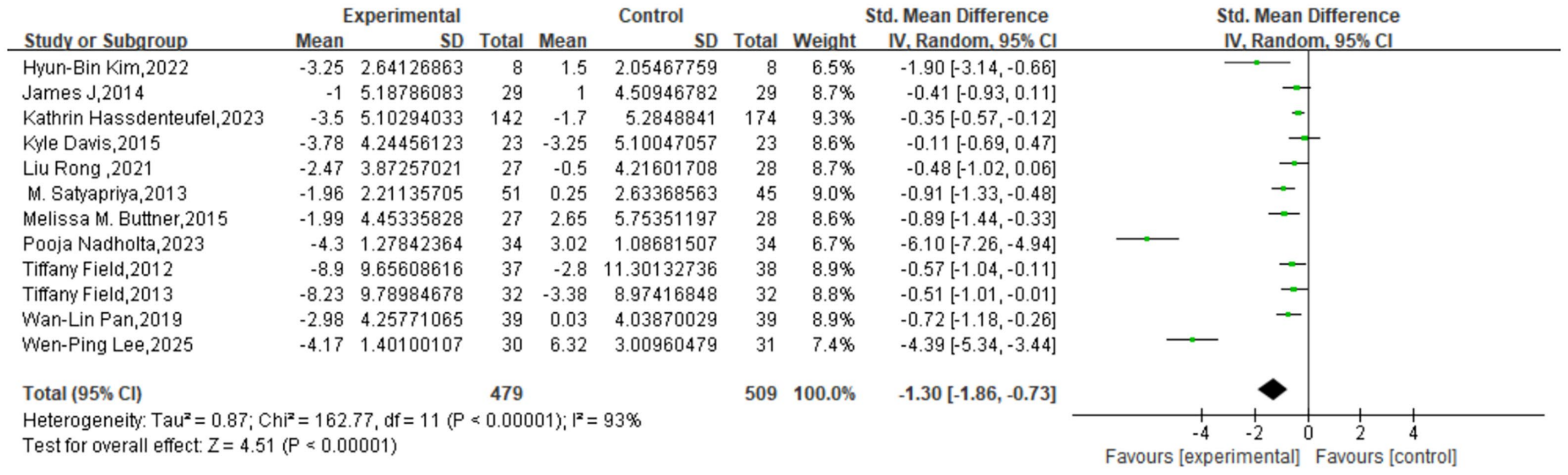
Meta-analysis results of depression.

The meta-analysis, incorporating 12 studies with a total of 988 participants, revealed a significant, large effect of mind–body interventions on depressive symptoms. The pooled effect size was an SMD of −1.30 (95% CI: −1.86 to −0.73, *p* < 0.00001), indicating a statistically significant advantage for the intervention group over the control group. However, a very high degree of heterogeneity was observed across the studies (*I*^2^ = 93%), suggesting substantial variation in effect sizes. This variability may be attributable to differences in population characteristics, intervention protocols, measurement tools, or study quality, warranting a cautious interpretation of the pooled estimate (Figure 3).

**Figure 4:**
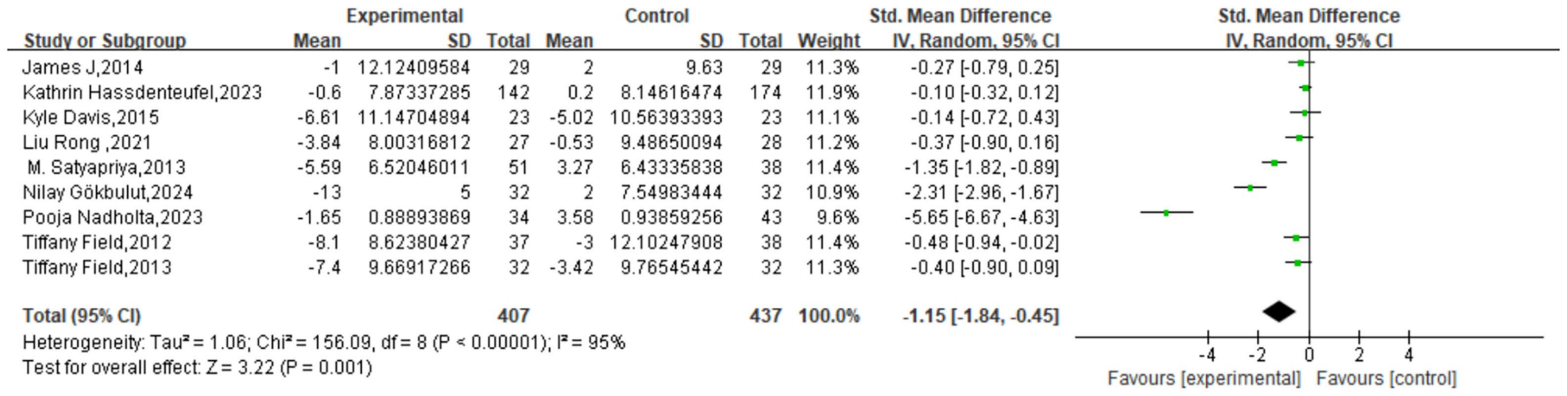
Meta-analysis results of anxiety.

Similarly, the meta-analysis of anxiety outcomes, which included 9 studies with 844 participants, demonstrated a significant, large effect in favor of the intervention. The pooled effect size was an SMD of −1.15 (95% CI: −1.84 to −0.45, *p* = 0.001). As with the depression outcome, very high heterogeneity was present (*I*^2^ = 95%). This substantial variability likely stems from a combination of factors, including differences in study design, participant characteristics, intervention modalities, assessment tools, and implementation settings. Consequently, further investigation through subgroup and sensitivity analyses is necessary to explore the sources of heterogeneity and to confirm the robustness of this finding (Figure 4).

To assess the robustness of our findings, a leave-one-out sensitivity analysis was conducted to evaluate the influence of each individual study on the overall pooled effect size. The analysis identified the study by Nadholta et al. (2023) as a potential outlier (28). Methodological concerns for this study included a notable worsening of symptoms in the control group, an exceptionally high intervention dosage (five sessions/week for 16 weeks), and the potential for significant un-controlled confounding variables. A subsequent sensitivity analysis was performed excluding this study. For the depression outcome, its removal reduced the heterogeneity from *I*^2^ = 93% to *I*^2^ = 87% and shifted the pooled effect size from an SMD of −1.30 (95% CI: −1.86, −0.73) to −0.89 (95% CI: −1.31, −0.48). The effect remained statistically significant (*p* < 0.001).

Notably, even after the exclusion of this outlier, the level of heterogeneity remained high (*I*^2^ = 87%). This is likely attributable to the combined influence of several factors. First, there was significant heterogeneity in the interventions themselves: yoga (eight studies), mindfulness (three studies), Pilates (one study), and Tai Chi Yoga (one study) may operate through distinct physiological and psychological mechanisms. Second, intervention parameters varied substantially, with session durations ranging from 20 to 180 min, program lengths from 4 to 16 weeks, and frequencies from one to five times per week. Third, the assessment tools were inconsistent; while scales such as the EPDS, CES-D, and PHQ-9 all measure depression, they differ in their sensitivity and specificity. Fourth, participant characteristics were diverse, with mean ages spanning 15.3 years (24.4 to 39.7) and a heavy focus on prenatal populations (92.3%, 12/13 studies), creating a dearth of postpartum data. Finally, sample sizes varied 20-fold (from 16 to 316), and the instability inherent in smaller studies may have amplified the overall heterogeneity. Therefore, these findings should be interpreted with caution, and clinicians should consider the specific intervention context and target population. While the sensitivity analysis confirms that the overall positive effect is not dependent on a single study, the high heterogeneity limits the interpretability and generalizability of the results.

### 3.5 Subgroup analysis

**Figure 5:**
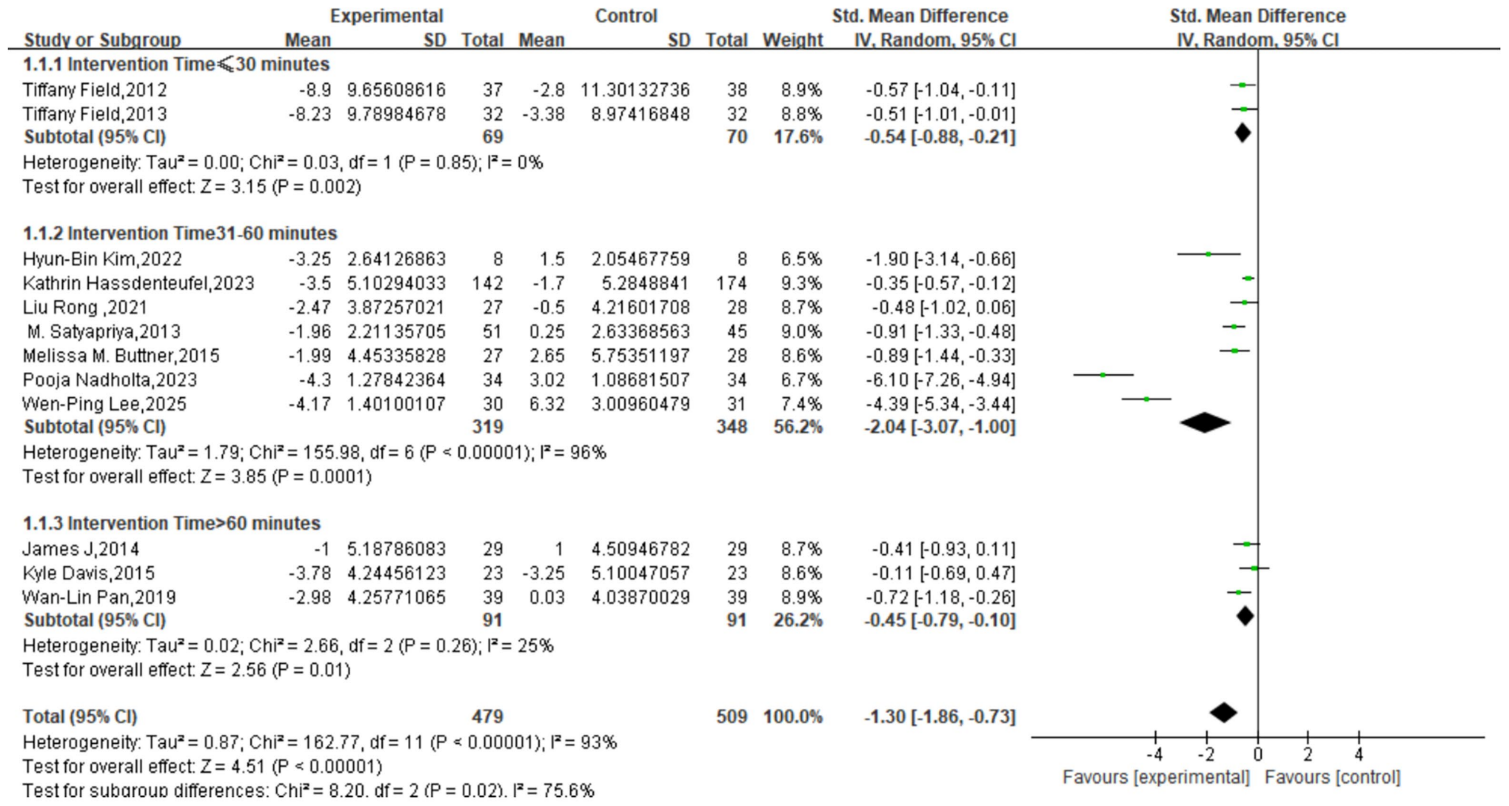
Subgroup analysis based on intervention time.

For the depression outcome, subgroup analysis based on session length revealed a statistically significant difference between subgroups (*χ*^2^ = 8.29, *p* = 0.02, *I*^2^ = 75.9%). All three subgroups demonstrated significant intervention effects: the short-duration group (≤30 min) yielded a pooled SMD of −0.54 (95% CI: −0.88, −0.21; *p* = 0.002) with no heterogeneity (*I*^2^ = 0%); the moderate-duration group (31–60 min) had a pooled SMD of −1.86 (95% CI: −2.76, −0.95; *p* < 0.0001) with very high heterogeneity (*I*^2^ = 96%); and the long-duration group (>60 min) showed a pooled SMD of −0.45 (95% CI: −0.79, −0.10; *p* = 0.01) with low heterogeneity (*I*^2^ = 25%) (Figure 5).

**Figure 6:**
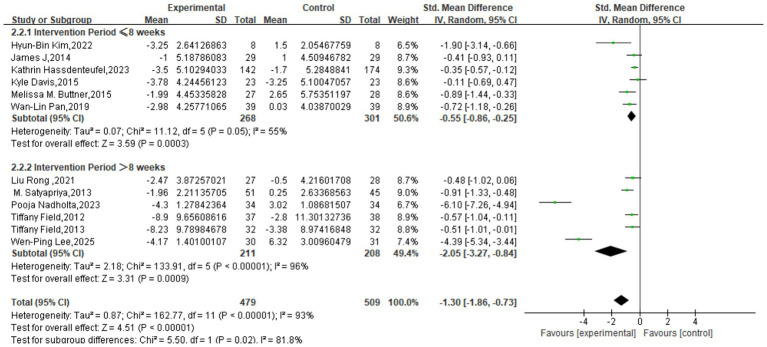
Subgroup analysis based on intervention period.

Subgroup analysis by program duration also showed a significant difference between groups (*χ*^2^ = 5.50, df = 1, *p* = 0.02, *I*^2^ = 81.8%). Programs of ≤8 weeks had a pooled SMD of −0.55 (95% CI: −0.86, −0.25) with moderate heterogeneity (*I*^2^ = 55%), while programs >8 weeks yielded a substantially larger effect with a pooled SMD of −2.05 (95% CI: −3.27, −0.84), albeit with high heterogeneity (*I*^2^ = 96%). Both subgroups showed a significant effect on depressive symptoms (*p* < 0.00001), suggesting that a longer program duration is a significant moderator of the intervention’s efficacy (Figure 6).

**Figure 7:**
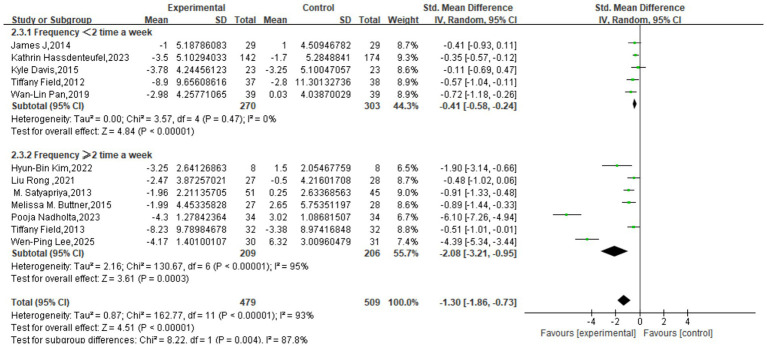
Subgroup analysis based on frequency.

Similarly, analysis based on intervention frequency indicated a significant difference between subgroups (*χ*^2^ = 8.22, df = 1, *p* = 0.004, *I*^2^ = 87.8%). The group with a frequency of <2 sessions/week had a pooled SMD of −0.41 (95% CI: −0.58, −0.24) with low heterogeneity (*I*^2^ = 0%; p < 0.00001). The group with ≥2 sessions/week showed a much larger effect with a pooled SMD of −2.08 (95% CI: −3.21, −0.95), though with very high heterogeneity (*I*^2^ = 95%; *p* = 0.0003). This indicates that intervention frequency is also a significant moderator of the effect on depression (Figure 7).

Subgroup analyses partially explained the heterogeneity for the depression outcome. Stratification by intervention parameters substantially reduced heterogeneity in several subgroups: it was eliminated (*I*^2^ = 0%) in the short session-length (≤30 min) and low-frequency (<2x/week) groups, and reduced to low (*I*^2^ = 25%) in the long session-length (>60 min) group and moderate (*I*^2^ = 55%) in the short program-duration (≤8 weeks) group. This suggests relative homogeneity among studies within these specific parameter sets. However, the subgroups associated with the largest effects (31–60 min sessions, >8 week duration, ≥2x/week frequency) retained very high levels of heterogeneity (*I*^2^ = 95–96%), implying that these categories may encompass diverse intervention protocols with widely varying effects.

For the anxiety outcome, none of the pre-specified moderators resulted in statistically significant subgroup differences. In the analysis by session length, the difference between subgroups was not significant (*p* = 0.06). For program duration, no significant difference was observed between the ≤8 weeks (SMD = −0.68) and >8 weeks (SMD = −1.57) groups (*p* = 0.30). Likewise, the difference between the <2 sessions/week (SMD = −0.46) and ≥2 sessions/week (SMD = −2.14) groups was not significant (*p* = 0.08). While the overall pooled effect for anxiety remained significant (SMD = −1.15, 95% CI: −1.84, −0.45; *p* = 0.001), the heterogeneity remained very high (*I*^2^ = 95%). These results suggest that session length, program duration, and frequency were not significant moderators of the intervention’s effect on anxiety in this analysis.

### 3.6 Publication bias

**Figure 8:**
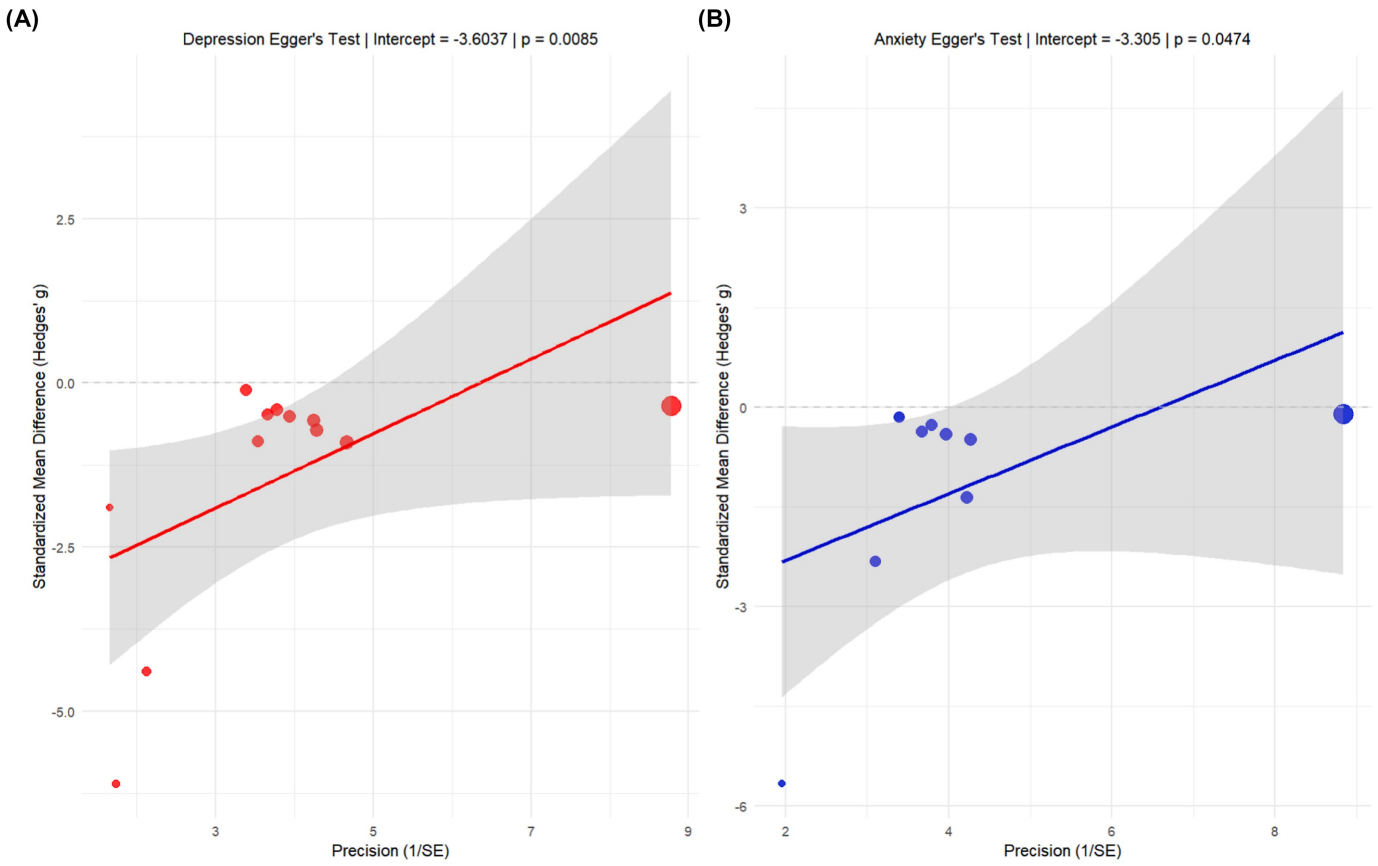
Egger test publication bias analysis chart. **(A)** Egger’s test for studies on depression (intercept = −3.6037, *p* = 0.0083). **(B)** Egger’s test for studies on anxiety (intercept = −3.305, *p* = 0.0474). The y-axis represents the standardized effect size (standardized mean difference/SE), and the x-axis represents precision (1/SE). Solid circles represent individual studies, the solid line is the weighted regression line, and the shaded area is the 95% confidence interval. The significant deviation of the regression line from zero in both analyses suggests potential publication bias.

Potential publication bias was assessed by examining funnel plot asymmetry using Egger’s regression test. For studies reporting on depression, Egger’s test indicated significant asymmetry (intercept = −3.6037, *p* = 0.0083), suggesting the presence of publication bias. For studies on anxiety, the test also detected a marginally significant asymmetry (intercept = −3.305, *p* = 0.0474). In both analyses, the distribution of studies on the precision-effect size plot was asymmetrical, with the regression line deviating significantly from the origin. These findings suggest a systematic difference in effect sizes between smaller and larger studies, which could reflect a tendency for studies with negative or small effects to remain unpublished. However, as Egger’s test can yield false-positive results when the number of included studies is small or heterogeneity is high, this interpretation must be made with caution (Figure 8).

**Figure 9:**
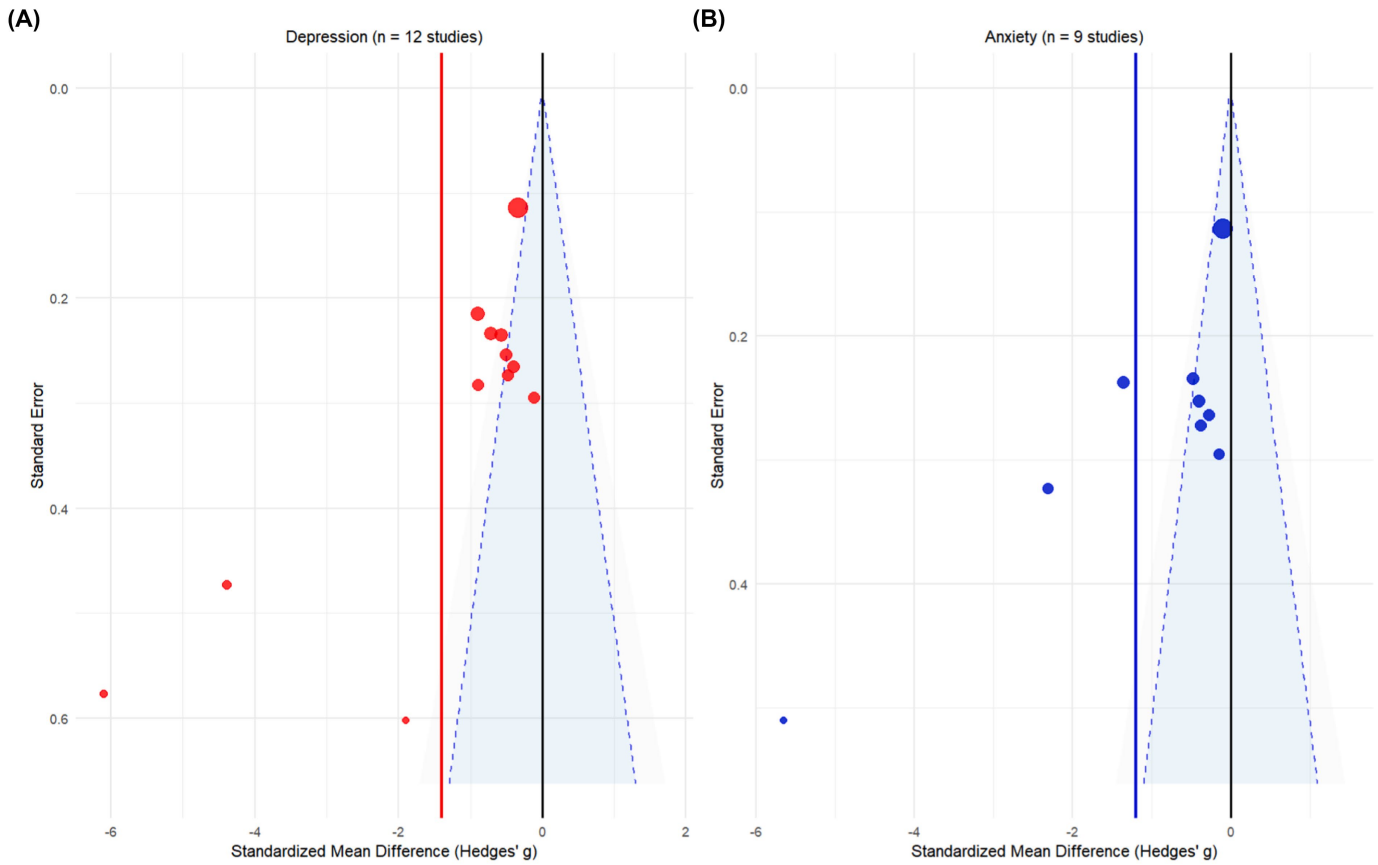
Analysis results of cutting and patching method. **(A)** Trim and fill analysis for studies on depression. **(B)** Trim and fill analysis for studies on anxiety. The solid circles represent the original studies included in the meta-analysis. The x-axis is the standardized mean difference (Hedges’ *g*), and the y-axis is the standard error (SE). The vertical line represents the pooled effect estimate, and the dashed lines form the 95% confidence interval funnel. Both analyses report zero imputed studies, indicating that no missing studies were detected that would require adjustment.

To further investigate the potential impact of publication bias, a sensitivity analysis was conducted using the trim and fill method. This analysis did not identify any missing studies to be imputed for either the depression or the anxiety outcomes (number of imputed studies = 0 for both). This result suggests that while Egger’s test detected asymmetry, it was not substantial enough to warrant adjustment of the pooled effect size by imputing hypothetical studies. The funnel plots generated by the trim and fill method showed that the observed studies were distributed relatively symmetrically around the pooled effect estimate. The discrepancy between the Egger’s test and trim and fill results may arise because the latter has a higher threshold for detecting bias, or because the observed asymmetry is a product of true between-study heterogeneity rather than publication bias. Taken together, while the possibility of publication bias cannot be entirely dismissed, its substantive impact on the overall results of this meta-analysis is likely limited (Figure 9).

### 3.7 Quality of evidence assessment

The quality of the evidence for each outcome was evaluated using the Grading of Recommendations Assessment, Development and Evaluation (GRADE) methodology, which classifies evidence as “high, moderate, low, or very low” (38). The initial quality of evidence from randomized controlled trials was considered high and was downgraded based on five domains: ① Risk of bias: Downgraded by one level if the overall risk of bias was rated as having “some concerns,” and by two levels if “high.” ② Inconsistency: Downgraded by one level for moderate or substantial heterogeneity (*I*^2^ > 25%) and by two levels for high heterogeneity (*I*^2^ > 75%). ③ Indirectness: Downgraded by one level if there were significant differences between the study populations, interventions, comparisons, or outcomes and the research question. ④ Imprecision: Downgraded by one level if the results were not statistically significant. ⑤ Publication bias: Downgraded by one level if Egger’s test was significant (*p* < 0.05). The GRADE assessment was performed independently by two authors, with the results summarized in Table 4.

**Table 4:** GRADE-based evidence quality assessment for study conclusions.

Outcome	No. of participants (studies)	GRADE assessment factors					Pooled effect [SMD (95% CI)]	Quality of evidence (GRADE)
		Risk of bias	Inconsistency	Indirectness	Imprecision	Publication bias		
Depressive symptoms	959 (12RCT)	Serious	Serious	Not serious	Not serious	Serious	−1.32[−1.93,-0.71]	⊕○○○Very low
Anxiety symptoms	856 (9RCT)	Serious	Serious	Not serious	Not serious	Serious	−1.14[−1.84,-0.44]	⊕○○○Very low

## 4 Discussion

### 4.1 Summary of evidence

The pooled effect size of this meta-analysis indicates that mind–body exercise interventions can statistically significantly improve depressive and anxiety symptoms in perinatal women. This result preliminarily suggests the potential of this non-pharmacological intervention in the field of perinatal mental health. However, the interpretation of this pooled effect size must be approached with caution. This analysis reveals two limiting factors in the current evidence base: significant statistical heterogeneity and a low level of evidence quality. First, the extremely high *I*^2^ values indicate significant differences in effect sizes among the included studies, with variation exceeding the scope of random error. This suggests that the observed positive effect is not a homogeneous result, and its generalizability is limited. Therefore, it is necessary to further clarify the specific conditions, applicable populations, and optimal intervention models for this intervention to be effective. This variation in effect size may stem from differences in intervention protocols, implementation parameters, and the baseline characteristics and socio-cultural backgrounds of the study subjects.

To explore the sources of heterogeneity, this study conducted subgroup analyses. The results showed that for the improvement of depressive symptoms, the duration and frequency of the intervention were important moderating factors. In contrast, the improvement of anxiety symptoms was not significantly associated with these parameters. Although the subgroup analyses provided some explanation, a high degree of heterogeneity remained within most subgroups, indicating the presence of other unidentified effect moderators. Finally, according to the GRADE system rating, the evidence quality for the two main outcomes of this study was “very low.” This rating is a composite of three factors: the high risk of bias prevalent in the included studies, significant inconsistency between studies, and detected publication bias. Therefore, although the pooled effect size shows a positive trend numerically, its evidence base is not yet solid, which limits the certainty and generalizability of the conclusion.

### 4.2 Comparison with previous research and mechanistic analysis

This study found that mind–body exercise has a substantial improvement effect on both perinatal depression and anxiety symptoms. This conclusion is consistent with some previous research findings but also has differences. For example, a meta-analysis by Wang et al. also reported a significant positive effect of yoga on perinatal depression and anxiety, with an effect size direction consistent with our findings (20). However, other studies have reached more complex conclusions (39). A review by Lever Taylor et al. found that mindfulness interventions had a moderate to large effect on perinatal anxiety but inconsistent effects on depressive symptoms (16). Furthermore, a study by Lin et al. pointed out that the antidepressant effect of yoga might be limited to women with pre-existing depressive symptoms at baseline (10). The discrepancies between these studies may be due to several factors. First, previous studies often focused on a single form of intervention, whereas this study evaluated various mind–body exercises as a single category, possibly combining the effects of different interventions. Second, the baseline mental health level of the subjects is an important moderating variable; the diverse population included in this study may have masked differential effects in specific subgroups. The findings of this study provide more comprehensive evidence for the application of mind–body exercise in perinatal mental health, but also suggest that its effect is not constant and is influenced by both the type of intervention and the characteristics of the population.

The psychological improvement observed in this study can be explained by potential mechanisms at both physiological and psychological-behavioral levels. It must be emphasized that since none of the included primary studies reported physiological indicators, the following discussion on physiological mechanisms is mainly based on theoretical deduction. From a physiological perspective, mind–body exercise may exert its effects by regulating the neuroendocrine and autonomic nervous systems. Theoretically, regular practice could influence the function of the hypothalamic–pituitary–adrenal (HPA) axis, regulating levels of stress hormones such as cortisol (40). However, the applicability of this speculation in the perinatal population needs to be considered cautiously, as pregnancy itself involves complex hormonal changes (41). Meanwhile, there is evidence that mind–body exercise can improve the balance of autonomic nervous function and increase heart rate variability (HRV) (42). Compared to physiological mechanisms, there is more observable evidence for psychological-behavioral mechanisms. The interventions included in this study (such as yoga and mindfulness) commonly incorporate elements that enhance body awareness and emotion regulation (43). Furthermore, regular practice itself can act as a behavioral activation strategy, combating feelings of helplessness in a depressive state by fostering a sense of mastery and accomplishment (44). However, it should be noted that the effect sizes found in this study showed extreme variation between studies, suggesting that the strength of these mechanisms may be regulated by multiple factors (45). The results of our subgroup analysis provide some clues. We found that the improvement of depressive symptoms was significantly correlated with the frequency and duration of the intervention, while this was not the case for anxiety symptoms. This may suggest that for depressive symptoms, the cumulative effects of regular practice (such as behavioral activation, cognitive reappraisal) are key; whereas the relief of anxiety symptoms may depend more on the immediate relaxation and emotion regulation skills acquired during practice, rather than a specific “dose.” Of course, this explanation remains speculative, and future research needs to incorporate multi-dimensional indicators and mediation analyses to more precisely reveal the core mechanisms by which mind–body exercise works in the perinatal period.

### 4.3 Clinical significance and evidence-based recommendations

The large effect sizes observed in this meta-analysis for both depression (SMD = −1.30) and anxiety (SMD = −1.15) suggest that mind–body exercise may be a potent non-pharmacological option for perinatal mental health. However, these findings must be interpreted in the context of the “very low” quality of evidence rating from the GRADE assessment for both primary outcomes. This rating was a result of downgrading for three critical factors: high risk of performance bias due to the lack of participant blinding across all studies (downgraded one level), very serious inconsistency due to extremely high statistical heterogeneity (*I*^2^ > 93%, downgraded two levels), and serious risk of publication bias (Egger’s test *p* < 0.05, downgraded one level). A “very low” quality rating implies that the true effect may be substantially different from the estimated effect, and thus any estimate is subject to a high degree of uncertainty.

In clinical practice, mind–body exercise may be considered as an adjunct to a comprehensive management plan for perinatal women who prefer non-pharmacological approaches, have concerns about medication side effects, or have contraindications. These interventions should not be considered a substitute for established, evidence-based treatments, particularly for individuals with moderate-to-severe symptoms. The preliminary findings from our subgroup analyses may offer some guidance for tailoring interventions: for women with predominantly depressive symptoms, higher frequency (≥2 sessions/week) and longer duration (>8 weeks) protocols were associated with larger effects, though this requires validation in higher-quality trials. For anxiety, the optimal intervention parameters remain unclear. In resource-limited settings, sessions of moderate duration (31–60 min) may offer a balance between feasibility and efficacy, but this suggestion remains tentative.

When implementing mind–body exercise, healthcare professionals should consider the individual’s physical health and gestational stage, prior exercise experience, cultural preferences, and issues of accessibility and cost. A thorough assessment is recommended before commencing any program, which should be conducted under professional guidance to ensure safety and appropriate modifications. A system for regular monitoring of symptoms and potential adverse effects should also be established. Importantly, these results should not be interpreted as evidence that all forms of mind–body exercise are effective for all perinatal women. Clinical decisions must be individualized, integrating patient preference, clinical judgment, and the best available evidence. Until higher-quality evidence is available, mind–body exercise should be positioned as a potential adjunctive option for perinatal mental health management, not as a first-line or standalone therapy.

### 4.4 Limitations and future directions

This study has multiple limitations that are intertwined and collectively affect the reliability and generalizability of the results. First, there is extremely high heterogeneity among the included studies, suggesting significant variation in effect sizes. Second, all studies had a high risk of bias in the blinding of participants and implementers, which may have introduced expectancy effects. Third, significant publication bias was detected, suggesting the selective non-publication of negative results. Fourth, the lack of objective physiological measures limits the understanding of the underlying mechanisms. Fifth, the sample sizes are relatively limited, and the studies are mainly concentrated on the prenatal period, with insufficient postpartum data. Sixth, the diversity of intervention forms, implementation protocols, and measurement tools complicates the interpretation of the results. Finally, the GRADE assessment showed the evidence quality is “very low,” severely limiting the certainty of clinical recommendations. Based on the current findings and limitations, future research should be deepened and improved in the following directions.

Based on these limitations, future research should proceed in several key directions. To address heterogeneity, more rigorous and standardized protocols are needed, including uniform intervention delivery, training standards, and quality control. Large-scale, multicenter RCTs with detailed, pre-specified plans for subgroup analyses (e.g., by baseline symptom severity, demographics) are recommended. To mitigate the inherent challenges of blinding, researchers should explore innovative designs using attention-matched or active control groups (e.g., light stretching) to partially control for expectancy. While participant blinding may be impossible, ensuring the blinding of outcome assessors is critical, and supplementing subjective reports with objective physiological or behavioral outcomes should be considered.

To elucidate the underlying mechanisms, future studies must systematically incorporate multi-level biomarkers, including neuroendocrine (e.g., cortisol rhythms, oxytocin), autonomic (e.g., heart rate variability), and inflammatory (e.g., IL-6, TNF-α) markers, alongside neuroimaging where feasible. This will not only help validate subjective improvements but also clarify the pathways through which these interventions work. Research should also expand to include a balanced representation of both prenatal and postpartum women, with extended follow-up periods (e.g., to one year postpartum) to assess long-term efficacy and preventive effects. Finally, implementation science studies are needed to evaluate the feasibility, acceptability, and cost-effectiveness of these interventions in real-world clinical settings.

### 4.5 Conclusion

The results of this systematic review and meta-analysis indicate that mind–body exercise interventions show potential for improving depressive and anxiety symptoms in perinatal women. However, the evidence base for this conclusion is not yet solid due to high heterogeneity between studies and a very low overall quality of evidence. In clinical application, this finding suggests that mind–body exercise can be considered a supplementary option, providing support for women who prefer non-pharmacological interventions or have mild symptoms, but it should not replace standard evidence-based treatments. To clarify its true efficacy, future research urgently needs to shift towards designing more rigorous, larger-sample randomized controlled trials, and using standardized intervention protocols and objective physiological indicators to elucidate the mechanisms of action. At the same time, it is recommended that policymakers consider integrating such non-pharmacological interventions into community maternal health care systems to improve their accessibility, and to increase funding for related high-quality research, thereby providing a more solid scientific basis for building a comprehensive perinatal mental health support strategy.

## Data Availability

The original contributions presented in the study are included in the article/Supplementary material, further inquiries can be directed to the corresponding author.
